# Comparison of miRNA Profiles of Cord Blood Stem Cells in Identical and Fraternal Twins

**DOI:** 10.22074/cellj.2019.5683

**Published:** 2018-11-18

**Authors:** Monireh Ajami, Mohammad Hadi Sadeghian, Masoud Soleimani, Mohammad Reza Keramati, Mansoureh Ajami, Azadeh Anbarlou, Amir Atashi

**Affiliations:** 1 Faculty of Medicine, Mashhad University of Medical Sciences, Mashhad, Iran; 2 Cancer Molecular Pathology Research Center, Mashhad University of Medical Sciences, Mashhad, Iran; 3 Department of Hematology, Faculty of Medical Sciences, Tarbiat Modares University, Tehran, Iran; 4 Department of Tissue Engineering, School of Advanced Technologies in Medicine, Shahid Beheshti University of Medical Sciences, Tehran, Iran; 5 Stem Cell and Tissue Engineering Research Center, Shahroud University of Medical Sciences, Shahroud, Iran

**Keywords:** Cord Blood, Epigenetic, Hematopoietic Stem Cells, miRNA, Twins

## Abstract

**Objective:**

The role of epigenetic in regulating of the gene expression profile the embryo has been documented. MicroRNAs (miRNAs) are one of these epigenetic mechanisms. Twins are valuable models in determining the relative contributions of genetics and the environment. In this study, we compared differences in the expression levels of 44 miRNAs in hematopoietic stem cells (HSCs) of identical twins to that of fraternal twins as a controls.

**Materials and Methods:**

In this experimental study, CD133^+^ HSCs were isolated from cord blood of identical and fraternal twins via magnetic-activated cell sorting (MACS). Variation in of gene expression levels of 44 miRNAs were evaluated using quantitative reverse transcription-polymerase chain reaction (qRT-PCR).

**Results:**

Significant differences in expression were observed in both fraternal and identical twins to varying degrees, but variations alteration in expression of the miRNAs were higher in fraternal twins.

**Conclusion:**

Identical twins had a positive correlation in miRNA expression, while the correlation was not statistically significant in fraternal twins. Altogether, more differences in miRNA expression level in fraternal twins can be attributed to the both genetics and the intrauterine environment. The contribution of the intrauterine environment and genetics to miRNAs expression in HSCs was estimated 8 and 92%, respectively. By comparing of miRNA expression in identical and fraternal twins and identification of their target genes and biological pathways, it could be possible to estimate the effects of genetics and the environment on a number of biological pathways.

## Introduction

MicroRNAs (miRNAs) are small (~22-nucleotide) noncoding RNA molecules that can negatively regulate gene expression at the post-transcriptional level (1, 2). miRNAs bind to their target mRNAs and cause instability and target mRNA fragmentation when this pairing is complete. In the case of a partial binding which often occurs in the 3´UTR, the mRNA is prevented from being translated into a protein. It is expected that each miRNA can regulate many mRNAs and each mRNA may be regulated by several miRNAs (3, 4). miRNA expression profiling is important because of key role of miRNA in regulating gene expression networks and their effects on many biological processes, as well as their role as disease markers (5, 6). 

Epigenetics refers to temporary modifications to DNA that can turn genes "on" or "off " (7). These modifications do not change the DNA sequence. Recent findings have shown the role of epigenetic mechanisms such as DNA methylation and histone modifications in miRNAs expression (8). A lot of research has shown that CpG islands upstream of miRNAs act as a promotersand are regulated through DNA methylation (9, 10). Enzymes which are involved in miRNA processing pathways also participate in epigenetic mechanisms (11). Some miRNAs participate in DNA methylation, for instance miR-165 and miR-166 are essential for PHABULOSA (PHB) methylation in Arabidopsis, alsothe key enzyme DNMT1, 3a and 3b are all potential targets for miRNAs (12, 13). In general, miRNAs can be considered an important factor in epigenetics and the control of gene expression (14).

Twins studies can provide information on the relative contribution of genetics and the environment on phenotypic characteristic and discover the etiology of the diseases. Recently, a study on twins has been done to assess the regulatory effects of epigenetic factors on gene expression (15). Differences in the epigenome can determine disease susceptibility in a pair of twins (16). Twins are considered a valuable model in determining the relative contribution of genetic and environmental factors regarding the relationship between epigenetics and miRNAs (17). Collection of umbilical cord blood (UCB) cells is considered a noninvasive method, and primitive CD133^+^ hematopoietic stem cells (HSCs) isolated from UCB would be appropriate for investigating of difference in the miRNA expression profiles of newborn twins. In this study, we compared and analyzed miRNA expression profiles of identical and fraternal twins. 

## Materials and Methods

### Subjects and samples

 This experimental study was approved by Ethical Committee of Mashad University of Medical Sciences (IR. MUMS.REC.1392.12). The study was performed using cord blood from two pairs of identical (monozygotic) and two pairs of fraternal (dizygotic) twins. Cord bloods were collected from 36-37 week full term twins with the informed consent of the mothers. Mothers were aged in the range of 30-35 years and the gender of both fraternal and identical twins was male.

### Zygosity

Same sex twins that shared a placenta with one or two amniotic sacs (monochorionic-diamniotic or monochorionic-monoamniotic) were considered as identical twins and same sex that come with two placentas and two amniotic sacs (dichorionic-diamniotic) were considered as fraternal twins.

### CD133^+^ cells isolation

UCB samples were obtained immediately after birth, diluted with hydroxyethyl starch in the ratio of 1:4 to deplete red blood cells. The diluted cell suspensions were gently layered over Ficoll-Paque (Pharmacia- Amersham, Piscataway, USA) and centrifuged for 20-30 minutes at 400×g at room temperature to the separate mononuclear cell fraction.

The samples were enriched for CD133^+^ cells with magnetic activated cell sorting (MACS) using CD133^+^ cell isolation Kit (MiltenyiBiotec, Gladbach, Germany) according to the manufacturer’s instructions. Purity of isolated CD133^+^ cells and the homogeneity of the population were assessed with flow cytometry.

### Flow cytometry analysis

Purity of isolated CD133^+^ cells from UCB using MACS and the homogeneity of the population were assessed by flow cytometry. CD133^+^ cells were stained with PE-conjugated anti-human CD133 antibody (Miltenyi Biotech, Germany) and mouse IgG1 antibody (IQ- Products, Netherlands) was used as an isotype control according to the manufacturer’s instructions. 

### RNA extraction and quantitative reverse transcription- polymerase chain reaction for miRNA

About 800.000 CD133^+^ cells were isolated from each bag of cord blood and 700.000 of them used for RNA extraction with TRIzol (Invitrogen, Carlsbad, CA, USA) according to themanufacturer’s instructions. cDNA synthesis was performedusing miRNA EasyScript cDNA Synthesis Kit (G269 ABM, USA following the manufacturer’s protocol. SynthesizedcDNA was mixed with primers and EvaGreen miRNA qPCRMasterMix-ROX (MasterMix-mR ABM, USA) followingthe manufacturer’s instructions. Quantitative reverse transcription-polymerase chain reaction (qRT-PCR) analysiswas performed on ABI 7000 system (Applied Biosystems, USA). Two reliable endogenous controls (U6–2, SNORD 48) were used to normalize and calculate the relative expressionlevels. Calculations were based on the comparative ..CTmethod. All of 46 primer pairs were custom-ordered fromABM Inc (Table S1) (See Supplementary Online Information at www.celljournal.org). All samples were run in triplicates.

### Bioinformatic analysis

DIANA-miRPath (http://diana.imis.athena-innovation.gr/ DianaTools/index.php?r=mirpath) was used to show whichbiological pathways are related to the miRNAs. DIANAmirExTra software (http://diana.cslab.ece.ntua.gr/hexamers/) was used to determine microRNA target genes.

### Estimating heritability

Studies on identical and fraternal twins provide an opportunity to estimate the contribution of the environment and genetics with the use of the heritability formula: H^2^=2 (r_mz_–r_dz_) H^2^: heritability/r: regression/mz: monozygotic/dz: dizygotic

### Statistical analysis

Statistical analyses were performed usingMicrosoft Excel. Data means were comparedusing Student’s t test and one-way ANOVA. Statistical significance was defined as P<0.05.

## Results

### Purity of CD133^+^ cells isolated from cord blood

**Fig.1:**
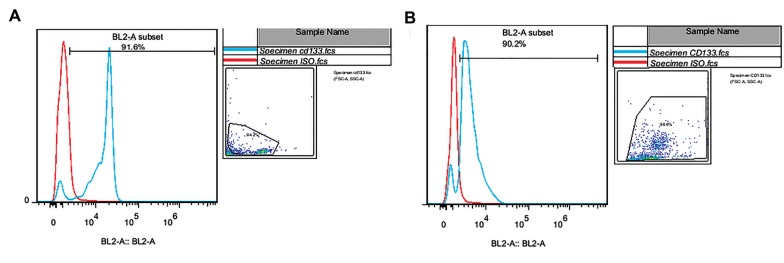
Flow cytometry result of CD133^+^ cells separated from cord blood of twins. A. Flow cytometry histogram for fraternal twins and B. Flow cytometry histogram for identical twins.

Cord blood samples were obtained from identical and fraternal twins. The purity of separated cells from the cord blood samples was measured for all of the twins. One flow cytometry histogram for fraternal twins (Fig .1A) and one for identical twins (Fig .1B) is presented here. Purity of CD133^+^ cells isolated from cord blood was about 90% in all samples. 

### miRNA expression profiling of CD133^+^ cells

Based to previous studies(18-24), 44 miRNAs that play keyroles in self renewal/differentiation and have high expressionin CD133^+^ HSCs from various origins (peripheral blood, bone marrow, and umbilical cord blood) were selected andevaluated (Table S1) (See Supplementary Online Informationat www.celljournal.org). The lists of MicroRNAs with thehighest expression in fraternal twins and identical twins arereported in Table 1. MiR-10b was not expressed in any of thesamples.

**Table 1:** MicroRNAs with the highest expression levels in fraternal twins and in identical twins (ΔCT)


Fraternal twins			
Pair 1		Pair 2	
miRNA	Level of expression	miRNA	Level of expression

*miR-107*	10.07	*miR-129-3P*	17.04
*miR-10a*	10.07	*miR-34c-3p*	17.01
*miR-20a*	9.77	*miR-181d*	16.08
*miR-411*	9.57	*miR-29a*	14.88
*miR-125d*	9.07	*miR-34b*	14.11
*miR-181d*	9.07	*miR-125d*	14.06
*miR-19b*	9.07	*miR-20a*	14.05
*miR-29a*	9.07	*miR-181b*	14.02
*miR-520h*	8.22	*miR-10a*	13.03
*miR-128*	8.22	*miR-181c*	13.03
*miR-144*	8.12	*miR-125a-3p*	13.02
*miR-34b*	8.12	*miR-34a*	13.00
*miR-142-5p*	8.10		
**Identical twins**			
*miR-181d*	10.82	*miR-181c*	12.20
*miR-20a*	10.82	*miR-144*	11.39
*miR-20b*	10.82	*miR-125a-3p*	11.36
*miR-29a*	10.82	*miR-181d*	10.40
*miR-107*	8.82	*miR-20a*	10.34
*miR-10a*	8.82	*miR-10a*	10.29
*miR-125d*	8.82	*miR-130a*	10.27
*miR-9*	7.32	*miR-29a*	10.05
*miR-106b*	6.12	*miR-19a*	9.80
*miR-19a*	5.82	*miR-181b*	9.45


**Fig.2:**
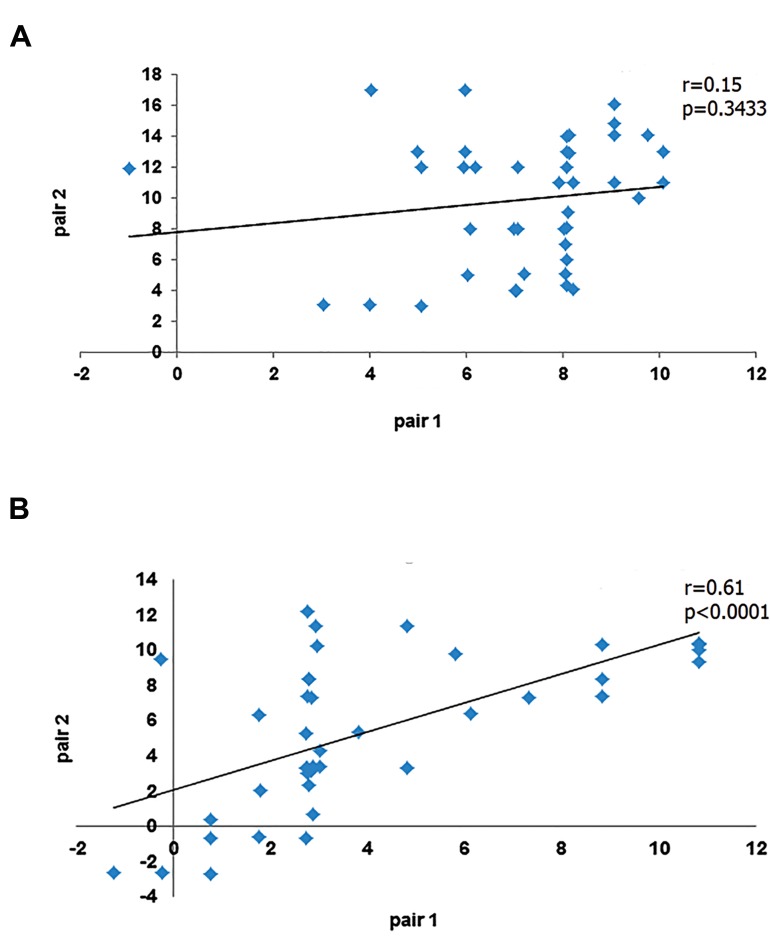
The Correlation between miRNAs expression. A. Fraternal twins and B. Identical twins.

There was not significant correlation between miRNA expression of pair 1 and pair 2 in fraternal twins (r=0.15, P=0.3433). There was a significant positive correlation between miRNA expression of pair 1 and pair 2 in identical twins (r=0.61, P<0.0001) (Fig .2).

**Fig.3:**
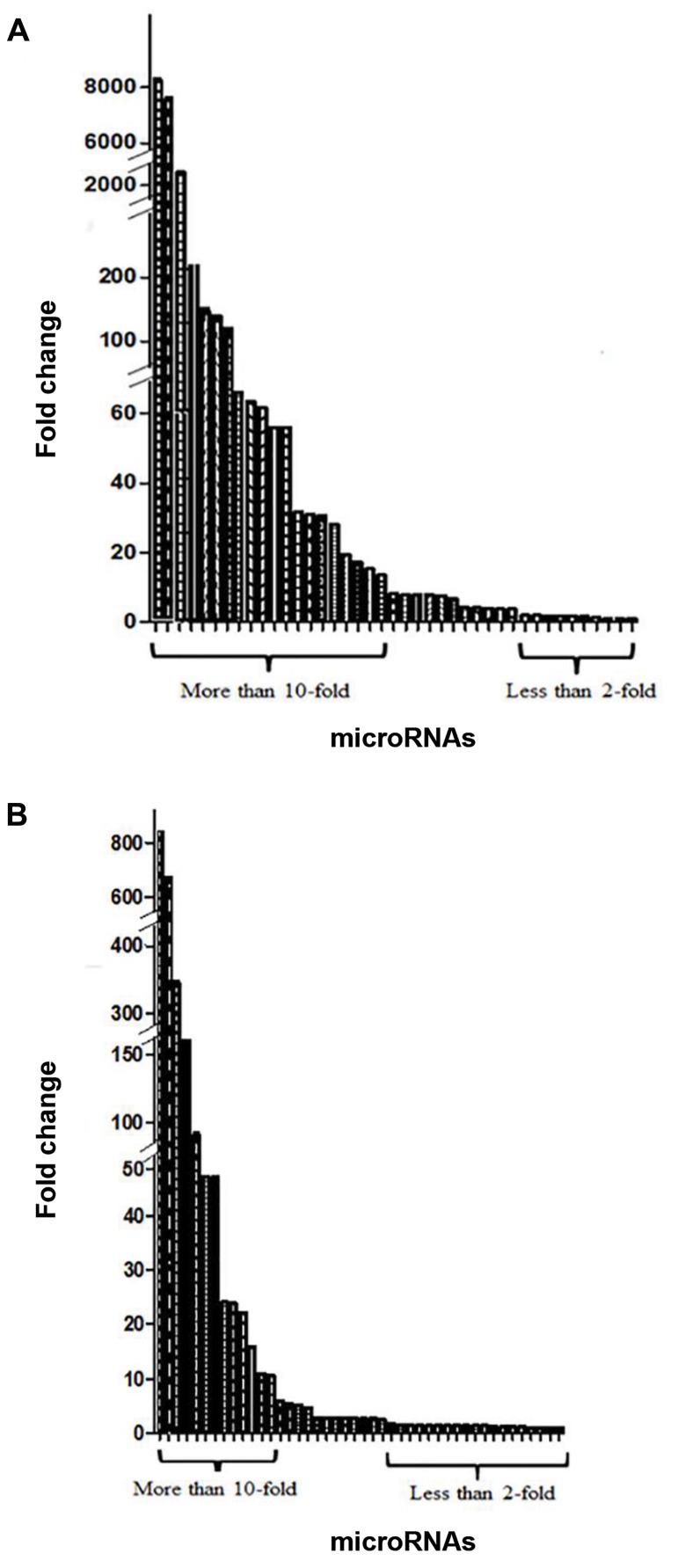
Bar graphs showing the difference in expression in studied of of 44 miRNAs. A. The levels of differential expression in fraternal twins and B. The levels of differential expression in identical twins.

The discordance of miRNA expression levels in identical and fraternal twins was calculated the using comparative Ct (..Ct) method of calculation. The mismatch variances in the levels of miRNA expression is shown in Table 2. Altogether 44 miRNA were categorized into three groups: high (more than 10fold), low (less than 2-fold) and moderate (between 2-10 fold) difference in expression. In fraternal twins, 20 miRNAs had high, 10 miRNAs had low and 13 miRNAs had moderate difference in expression. In identical twins, 13 miRNAs had high, 18 miRNAs had low and 12 miRNA had moderate difference in expression. MiR-10b was not expressed in any of the samples. The levels of differential expression in studied of the miRNAs in identical and fraternal twins are shown in Figure 3. These miRNAs can be divided in to four groups (A, B, C, and D) according to how their expression is affected by genetics and the environment (Table S2) (See Supplementary Online Information at www.celljournal.org) target genes and biological pathways related to these miRNAs are shown in Table 3 (heat maps are presented in Figure S1) (See Supplementary Online Information at www. celljournal.org). 

### Calculation of heritability

The role of genetic contribution in microRNAs expression levels was estimated at 92% [H_2_=2 (0.61– 0.15)=0. 92]. The role of environment on the differences in microRNA expression levels was estimated at 8% (10.92= 0.08). 

**Table 2:** The discordance of miRNA expression in identical and fraternal twins. High difference and low difference between miRNA expression


High difference miRNAs expression			
Fraternal twins		Identical twins	
Expression discordance (fold change)		Expression discordance (fold change)	

*miR-129-3P*	8248.98	*miR-181b*	837.53
*miR-106b*	7858.29	*miR-181c*	689.78
*miR-34c-3p*	2105.57	*miR-144*	352.13
*miR-34a*	261.37	*miR-130a*	158.68
*miR-125a-3p*	131.59	*miR-125a-3p*	93.05
*miR-181d*	128.89	*miR-519d*	47.17
*miR-17*	123.63	*miR-520h*	47.17
*miR-519d*	66.25	*miR-181a*	24.08
*miR-34b*	63.55	*miR-19b*	23.75
*miR-181b*	61.81	*miR-17*	22.16
*miR-181a*	56.10	*miR-19a*	15.77
*miR-29a*	56.10	*miR-92a*	10.92
*miR-125b*	31.77	*miR-24*	10.63
*miR-181c*	31.12		
*miR-19a*	30.69		
*miR-144*	28.24		
*miR-20a*	19.42		
*miR-520h*	17.38		
*miR-130a*	15.45		
*miR-34c-5p*	13.64		
**Low difference miRNAs expression**			
*miR-223*	1.97	*miR-29a*	1.70
*miR-155*	1.94	*miR-221*	1.47
*miR-107*	1.93	*miR-20a*	1.39
*miR-142-5p*	1.91	*miR-155*	1.38
*miR-411*	1.35	*miR-22*	1.38
*miR-221*	1.00	*miR-411*	1.37
*miR-92a*	1.00	*miR-181d*	1.33
*miR-10b*	1.00	*miR-16*	1.31
*miR-20b*	1.00	*miR-34c-3p*	1.31
*miR-93*	1.00	*miR-106b*	1.21
		*miR-34a*	1.20
		*miR-34b*	1.20
		*miR-34c-5p*	1.16
		*miR-9*	1.01
		*miR-129-3p*	1.00
		*miR-125b*	1.00
		*miR-10b*	1.00
		*miR-125a-5p*	1.00
		*miR-128*	1.00


**Table 3:** Target genes and biological pathways related to the miRNAs (group A: more affected by genetic, group


Group	miRNA	Target genes	KEGG pathway

A	*miR-129-3P*		Cell cycle
	*miR-106b*	ZNF419, RTN4, B2M, KLHL28	Chronic myeloid leukemia
	*miR-34c-3p*		Pathways in cancer
	*miR-34a*	ARHGAP1, KDRF(hsa), CDC46(hsa), ALR(hsa)	P53 signaling pathway
	*miR-34b*	MET, CREB, CDK4	HIF-1 signaling pathway
	*miR-29a*	E2F7, ACTB	PI3K-Akt signaling pathway
	*miR-125b*	CALU, EFNB2, RPA1	
	*miR-34c-5p*	MET, MYB, CDK4	
	*miR-20a*	RTN4, B2M, BICD2, GPR63	
B	*miR-181b*	TCL1, CDX2, BCL2	TGF-beta signaling pathway
	*miR-181c*	POLR2B, TWF1,CCNG1	
	*miR-144*	FGG, FGB	
	*miR-520h*	SMAD6, ABCG2	
	*miR-130a*	POLR2B, RTN4, TWF1	
C	*miR-181d*	BCL2	Pathways in cancer
	*miR-17*	RBM14, PTK4, SOX4, B2M, KLHL28, POU2F1	P53 signaling pathway
	*miR-519d*	PPARA, CDKN1A	PI3K-Akt signaling pathway
	*miR-181a*	FAM47B, POLR2B,TWF1	
	*miR-19a*	POLR2B, 2DHHC18, ESR1,TWF1	
	*miR-125a-3p*		
D	*miR-24*	SLITRK1, NOTUN, COPS7A, ABCB10, CCL2	Cell cycle
	*miR-19b*	POLR2B, ZDHHC18, ESR1	Chronic myeloid leukemia
	*miR-92a*	ANP32E, SAP18, ALKBH3, SOX4	P53 signaling pathway
			RNA transport
			TGF-beta signaling pathway


## Discussion

Studies on twins have provided the possibility of determining the contribution of genes and environment to phenotypic characteristics and etiology of diseases (15). Recently, studies on twins were performed to introduce epigenetic as a factor effecting gene expression (25). Differences in the epigenome can show the susceptibility to disease, variability in age of onset and severity of diseases in twins (16, 26).

Differences in the expression of some genes particularly in twins represent a group of genes whose expression levels are more sensitive to the effects of the environment. The lowest difference in the intrauterine environment can affect gene expression profile (27). The fetal programming is independent of genomic DNA sequences and may be associated with epigenetic mechanisms (28).

Identical twins are a good model for studying epigenetic differences. To date, conflicting evidence of epigenetic differences in identical twins from childhood to adulthood have been reported (29). In general, phenotypic discordance between identical twins is attributed to non- shared environments that identical twins in encounter during their lives (15, 29). The epigenome is dynamic and affected by environmental changes. Many studies have been shown that epigenetics is a key factor in the discordance between identical twins (30-32).

Recent studies have proposed some reasons for differences between identical twins. One of them is miRNAs which are able to control epigenetic mechanisms (33). On the other hand, epigenetic mechanisms are also capable of regulating miRNA expression (32). 

In this study, we compared miRNA expression levels in HSCs derived from cord blood of identical and fraternal twins at birth. The evaluation of miRNAs in both identical and fraternal twins showed different expression levels of miRNAs, to a greater extent in (fraternal twins than identical twins). So far, no similar study has investigated the differences in miRNA expression in cord blood HSCs. However, there have only been a few studies on the differences in methylation and genomic imprinting in identical twins (34).

Ollikainen et al. (35) evaluated the level of methylation in different tissues in identical and fraternal twins. They found differences in the methylation of specific loci in newborn twins. They attributed the epigenetic differences in identical twins to environmental factors and random events which occur in the uterus. But in fraternal twins, genetic diversity plays a major role. The difference in methylation in identical twins was different even between same tissues in twin pairs. 

Gordon et al. (27) compared gene expression in mononuclear and endothelial cells of UCB in identical twins. They observed significant differences in gene expression and concluded that these differences may be attributed to the intrauterine environment. Gordon et al. (36) also studied the methylation profile of CpG regions as a phenotype in different tissues (mononuclear and endothelial cells of UCB and endothelial cells of placenta) in twins. Identical twins had many differences at birth but differences were greater between fraternal twins.

In this study, the difference in the expression of 44 miRNAs which have high expression in cord blood stem cells were evaluated in two pairs of identical and fraternal twins. As previously mentioned these miRNAs divided in to four groups according to how their expression is affected by genetics and the environment. Group A contains miRNAs which showed high differences in expression in fraternal twins, but little difference in identical twins. This group is impacted to higher degree by genetics than the environment at the level of mRNA expression. Group B contains miRNAs which had high differences in both types of twins. The expression of miRNAs is likely more influenced by the environment. Group C is similar to group B, but the differences between fraternal twins were higher than between identical twins. Group D contains miRNAs which had high differences in expression in identical twins, but not in fraternal twins. In order to demonstrate the importance of these four groups, target genes and biological pathways were predicted. More than 80% of miRNAs in each group are involved in the mentioned biological pathways.

miRNAs in group A are involved in some pathways like hypoxia-inducible factor 1 (HIF-1) signaling. There for miRNAs related to the HIF-1 pathway are more affected by genetics, and the intrauterine environment has not been a major contributor in determining the expression levels of these miRNAs. Group B which is involved in the transforming growth factor-beta (TGF-beta) signaling pathway also showed a significant effect from the intrauterine environment in the regulation of their expression.

In groups C and D, both factors, environment and genetic, are involved in determining the level and discordant expression of miRNAs. Group C consists of miRNAs which had high differences in both identical and fraternal twins, but the differences in fraternal twins were higher than in identical twins. In fact, the combined effects of genetic variation and the environment plays an important role in increasing the variance in this group. miRNAs placed in group D showed high expression differences in identical twins, but had low discordance in fraternal twins despite them having genetic heterogeneity, meaning the effect of environment and the expression of these miRNAs is a little.

In summary, our study showed that observed discordance in miRNAs expression in identical twins can be attributed to the intrauterine environment (its contribution was estimated at 8%). In other words, miRNA expression levels can be affected by the smallest difference in intrauterine environment such as different position of the twins in the uterus. Expression discordance of the studied miRNAs was higher in fraternal twins than identical twins. In fraternal twins in addition to the environment, heterogeneous genetics has an important role (its contribution was estimated at 92%).

## Conclusion

The differences in the expression of 44 miRNAs which have high expression levels in cord blood stem cells were evaluated in two pairs of identical and fraternal twins. The identical twins had a positive correlation in miRNA expression, while the correlation was not statistically significant in fraternal twins. Altogether, more discordance in miRNA expression of fraternal twins can be attributed to both genetics and the intrauterine environment. The Contribution of the intrauterine environment and genetics on miRNA expression in HSCs was estimated at 8 and 92%, respectively. By comparing miRNAs expression levels in identical and fraternal twins and identifying their target genes and biological pathways, estimating the contribution of genetics and the environment to a number of biological pathways is possible.

## Supplementary PDF


