# Supplementary material for: Comparison of miRNA Profiles of Cord Blood Stem Cells in Identical and Fraternal Twins
Source: Cell J. 2018 Nov 18;21(1):78–85. doi: 10.22074/cellj.2019.5683 (PMC6275421; doi:10.22074/cellj.2019.5683)
Supplement: Supplementary file 1 [file Cell-J-21-78-S01.pdf]

www.celljournal.org

Supplementary Information for

Comparison of miRNA Profiles of Cord Blood Stem Cells in Identical and Fraternal Twins

Monireh Ajami, M.Sc.[1], Mohammad Hadi Sadeghian, M.D.[1, 2]*, Masoud Soleimani, Ph.D.[3]*, Mohammad Reza Keramati, M.D.[1, 2], Mansoureh Ajami, M.Sc.[3], Azadeh Anbarlou, M.Sc.[4], Amir Atashi, Ph.D.[5]

1. Faculty of Medicine, Mashhad University of Medical Sciences, Mashhad, Iran
2. Cancer Molecular Pathology Research Center, Mashhad University of Medical Sciences, Mashhad, Iran
3. Department of Hematology, Faculty of Medical Sciences, Tarbiat Modares University, Tehran, Iran
4. Department of Tissue Engineering, School of Advanced Technologies in Medicine, Shahid Beheshti University of Medical Sciences, Tehran, Iran
5. Stem Cell and Tissue Engineering Research Center, Shahroud University of Medical Sciences, Shahroud, Iran

**Corresponding Addresses: P.O.Box: 9176759416, Faculty of Medicine, Mashhad University of Medical Sciences, Mashhad, Iran*
P.O.Box: 14115-331, Department of Hematology, Faculty of Medical Sciences, Tarbiat Modares University, Tehran, Iran
Emails: sadeghianmh@mums.ac.ir, soleim_m@modares.ac.ir

Table S1: List of evaluated miRNAs and primers used for quantitative reverse transcription polymerase chain reaction (qRT-PCR)

Accession	miRNA/Gene	ABM Cat Number
MIMAT0000680	*hsa-miR-106b*	MPH01021
MIMAT0000104	*hsa-miR-107*	MPH01022
MIMAT0000253	*hsa-miR-10a*	MPH01023
MIMAT0000254	*hsa-miR-10b*	MPH01024
MIMAT0004602	*hsa-miR-125a-3p*	MPH01079
MIMAT0000443	*hsa-miR-125a-5p*	MPH01080
MIMAT0000423	*hsa-miR-125b*	MPH01081
MIMAT0000424	*hsa-miR-128*	MPH01110
MIMAT0004605	*hsa-miR-129-3p*	MPH01125
MIMAT0000425	*hsa-miR-130a*	MPH01142
MIMAT0000434	*hsa-miR-142-3p*	MPH01162
MIMAT0000433	*hsa-miR-142-5p*	MPH01163
MIMAT0000436	*hsa-miR-144*	MPH01165
MIMAT0000449	*hsa-miR-146a*	MPH01169
MIMAT0000646	*hsa-miR-155*	MPH01188
MIMAT0000068	*hsa-miR-15a*	MPH01189
MIMAT0000069	*hsa-miR-16*	MPH01191
MIMAT0000070	*hsa-miR-17*	MPH01192
MIMAT0000256	*hsa-miR-181a*	MPH01193
MIMAT0000257	*hsa-miR-181b*	MPH01194
MIMAT0000258	*hsa-miR-181c*	MPH01195
MIMAT0002821	*hsa-miR-181d*	MPH01196
MIMAT0000073	*hsa-miR-19a*	MPH01242
MIMAT0000074	*hsa-miR-19b*	MPH01243
MIMAT0000075	*hsa-miR-20a*	MPH01257
MIMAT0001413	*hsa-miR-20b*	MPH01258
MIMAT0000076	*hsa-miR-21*	MPH01259
MIMAT0000077	*hsa-miR-22*	MPH01277
MIMAT0000278	*hsa-miR-221*	MPH01281
MIMAT0000279	*hsa-miR-222*	MPH01282
MIMAT0000280	*hsa-miR-223*	MPH01283
MIMAT0000080	*hsa-miR-24*	MPH01294
MIMAT0000086	*hsa-miR-29a*	MPH01310
MIMAT0000255	*hsa-miR-34a*	MPH01453
MIMAT0004676	*hsa-miR-34b*	MPH01454
MIMAT0004677	*hsa-miR-34c-3p*	MPH01455
MIMAT0000686	*hsa-miR-34c-5p*	MPH01456
MIMAT0003329	*hsa-miR-411*	MPH01607
MIMAT0001631	*hsa-miR-451*	MPH01706
MIMAT0002853	*hsa-miR-519d*	MPH01782
MIMAT0002867	*hsa-miR-520h*	MPH01794
MIMAT0000441	*hsa-miR-9*	MPH02015
MIMAT0000092	*hsa-miR-92a*	MPH02020
MIMAT0000093	*hsa-miR-93*	MPH02022
-	*SNORD48*	MPH00005
-	*U6-2*	MPH00001

Table S2: Grouping of miRNAs based on the contribution of genetics and environmental effects

**Group**	**A**	**B**	**C**	**D**
**Cause of discordance**	**More effect of genetic**	**More effect of environment**	**The effect of environment and genetics in the same direction**	**The effect of environment and genetics in the opposite direction**
microRNA	*miR-129-3P*	*miR-181b*	*miR-181d*	*miR-24*
	miR-106b	*miR-181c*	*miR-17*	*miR-19b*
	miR-34c-3p	*miR-144*	*miR-519d*	*miR-92a*
	miR-34a	*miR-520h*	*miR-181a*	
	miR-34b	*miR-130a*	*miR-19a*	
	miR-29a		*miR-125a-3p*	
	miR-125b			
	miR-34c-5p			
	miR-20a			

A

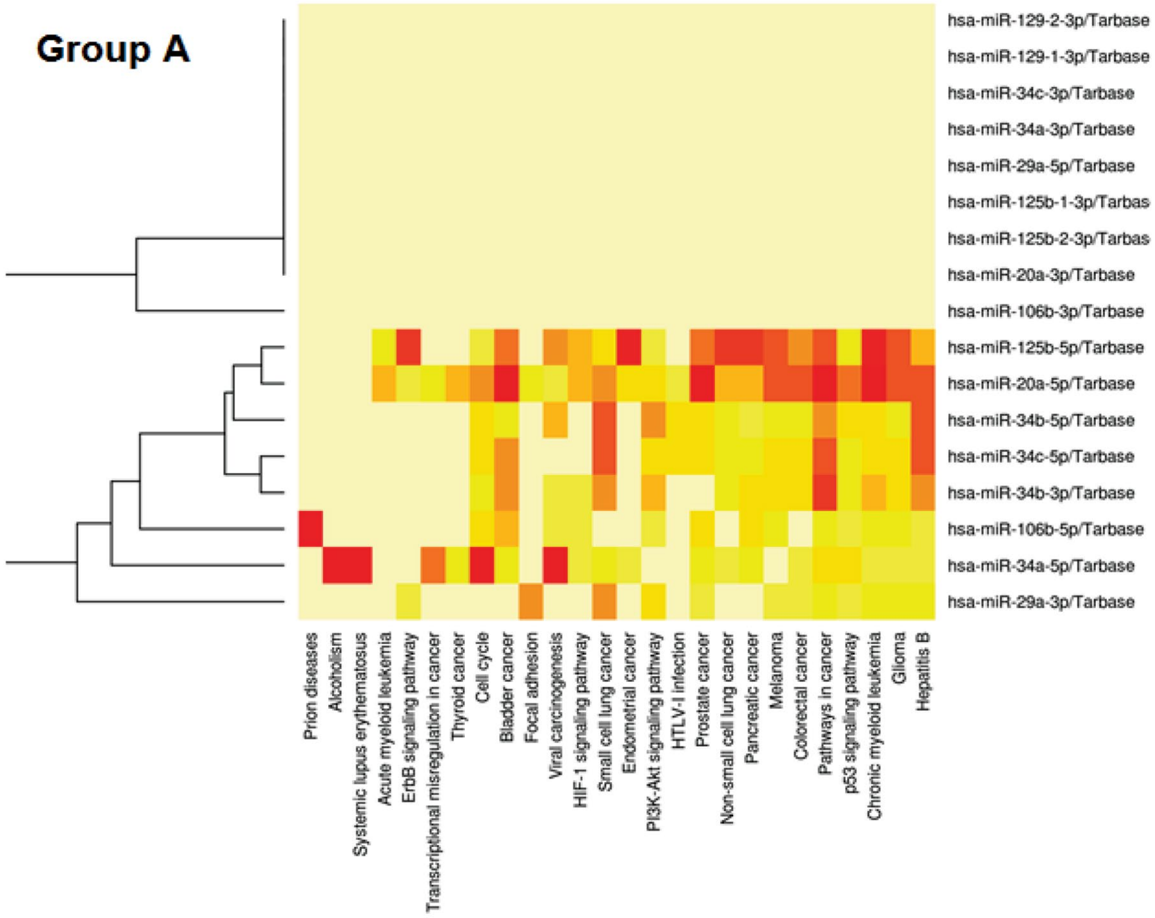

B

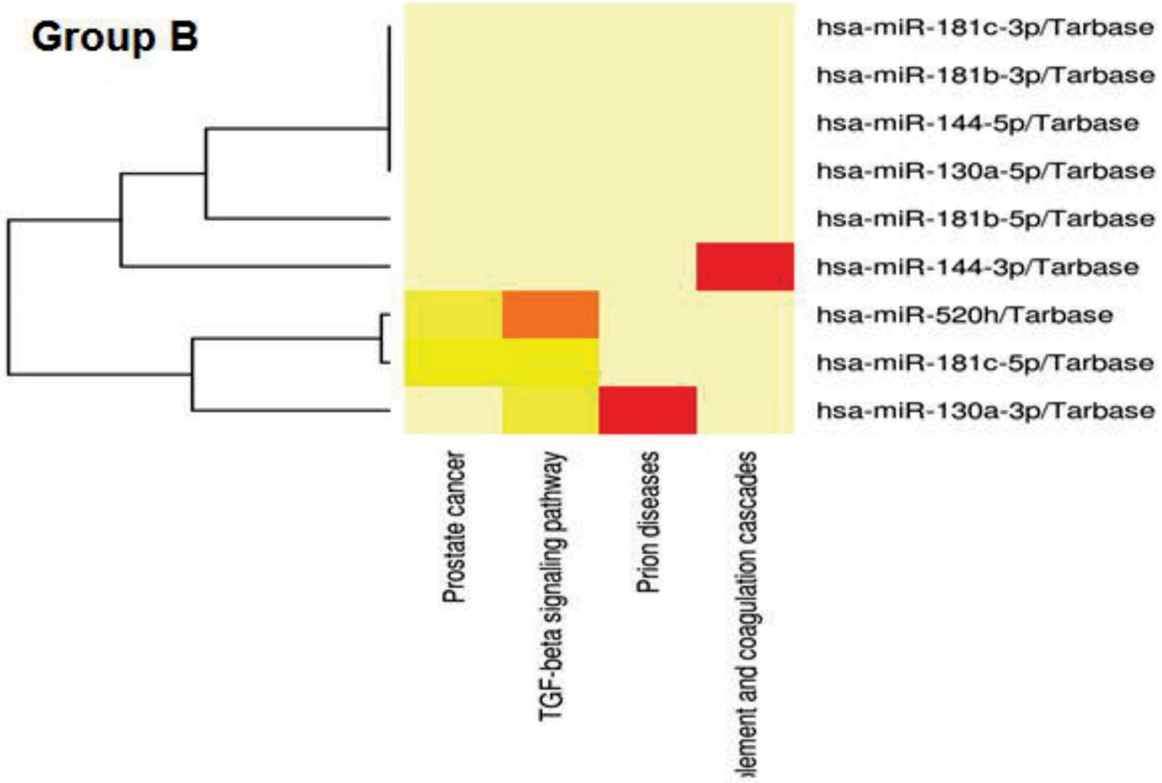
Group B
hsa-miR-181c-3p/Tarbase
hsa-miR-181b-3p/Tarbase
hsa-miR-144-5p/Tarbase
hsa-miR-130a-5p/Tarbase
hsa-miR-181b-5p/Tarbase
hsa-miR-144-3p/Tarbase
hsa-miR-520h/Tarbase
hsa-miR-181c-5p/Tarbase
hsa-miR-130a-3p/Tarbase
Prostate cancer
TGF-beta signaling pathway
Prion diseases
lement and coagulation cascades

C

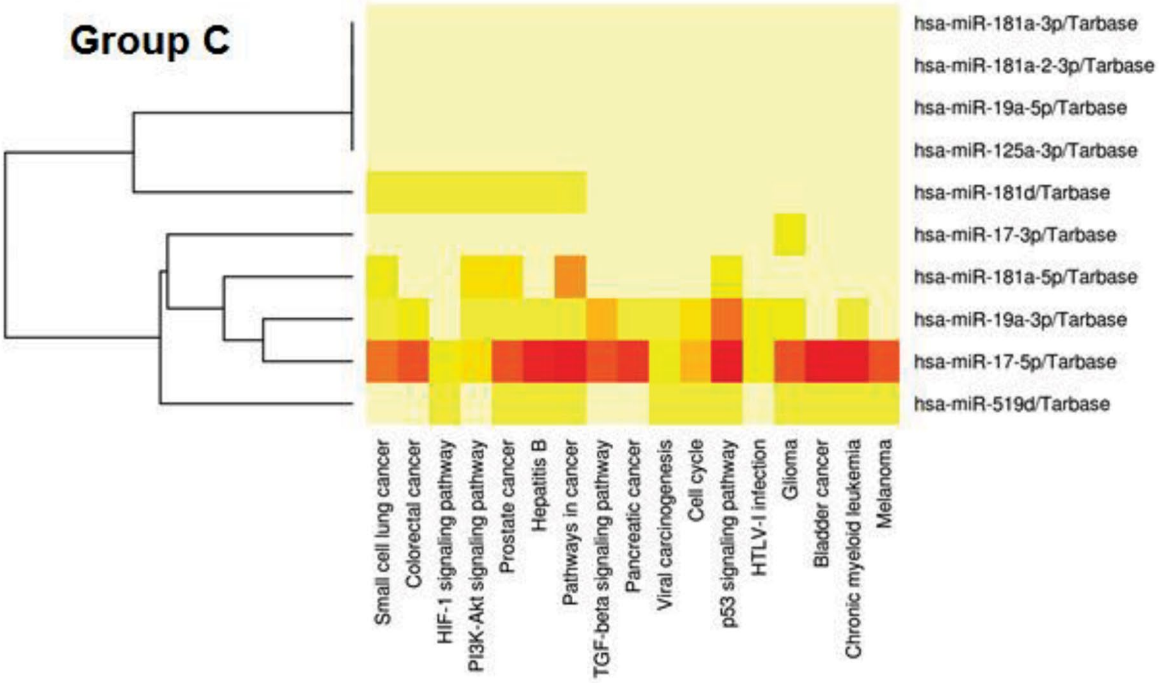
Group C
hsa-miR-181a-3p/Tarbase
hsa-miR-181a-2-3p/Tarbase
hsa-miR-19a-5p/Tarbase
hsa-miR-125a-3p/Tarbase
hsa-miR-181d/Tarbase
hsa-miR-17-3p/Tarbase
hsa-miR-181a-5p/Tarbase
hsa-miR-19a-3p/Tarbase
hsa-miR-17-5p/Tarbase
hsa-miR-519d/Tarbase
Small cell lung cancer
Colorectal cancer
HIF-1 signaling pathway
PI3K-Akt signaling pathway
Prostate cancer
Hepatitis B
Pathways in cancer
TGF-beta signaling pathway
Pancreatic cancer
Viral carcinogenesis
Cell cycle
p53 signaling pathway
HTLV-I infection
Glioma
Bladder cancer
Chronic myeloid leukemia
Melanoma

D

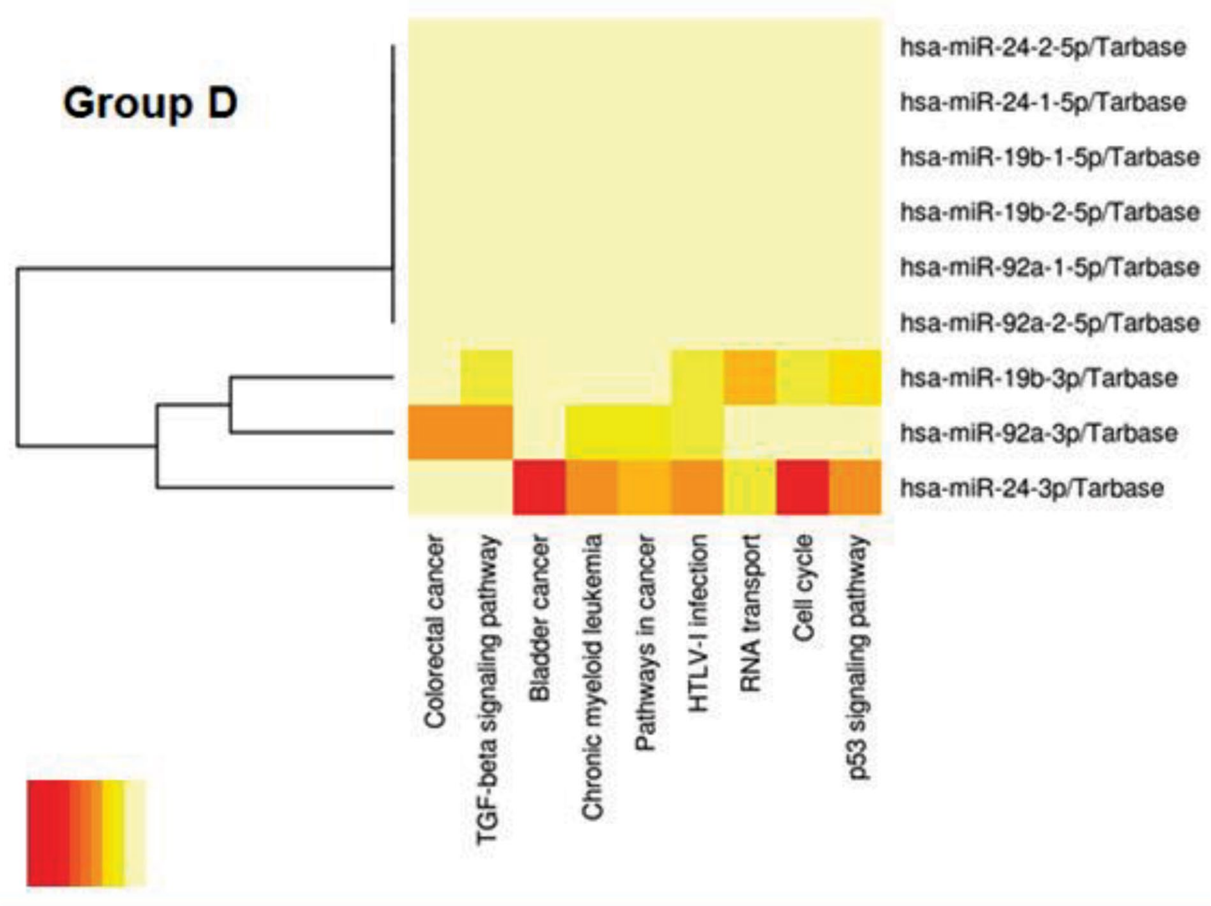

Fig.S1: Heat maps of all four groups. **A, B, C,** and **D.** Graphical data showing the involvement of miRNAs in biological pathways. Red color indicates high possibility, a reduction in color intensity indicates a decline in the possibility, contribution of the miRNA in the mentioned biological pathway.
